# Comparison of Myocardial Layer-Specific Strain and Global Myocardial Work Efficiency During Treadmill Exercise Stress in Detecting Significant Coronary Artery Disease

**DOI:** 10.3389/fcvm.2021.786943

**Published:** 2022-01-17

**Authors:** Jingru Lin, Lijian Gao, Jia He, Mengyi Liu, Yuqi Cai, Lili Niu, Ying Zhao, Xiaoni Li, Jiangtao Wang, Weichun Wu, Zhenhui Zhu, Hao Wang

**Affiliations:** ^1^Department of Echocardiography, National Center for Cardiovascular Diseases, Fuwai Hospital, Chinese Academy of Medical Sciences and Peking Union Medical College, Beijing, China; ^2^Department of Cardiology, National Center for Cardiovascular Diseases, Fuwai Hospital, Chinese Academy of Medical Sciences and Peking Union Medical College, Beijing, China; ^3^Cardiac Arrhythmia Center, National Center for Cardiovascular Diseases, Fuwai Hospital, Chinese Academy of Medical Sciences and Peking Union Medical College, Beijing, China; ^4^General Electric Healthcare, Beijing, China; ^5^Key Laboratory of Cardiovascular Imaging (Cultivation), Chinese Academy of Medical Sciences, Beijing, China

**Keywords:** myocardial work efficiency, layer-specific strain, speckle-tracking echocardiography, treadmill exercise stress, coronary artery disease

## Abstract

**Background:**

Myocardial layer-specific strain can identify myocardial ischemia. Global myocardial work efficiency (GWE) based on non-invasive left ventricular (LV) pressure-strain loops is a novel parameter to determine LV function considering afterload. The study aimed to compare the diagnostic value of GWE and myocardial layer-specific strain during treadmill exercise stress testing to detect significant coronary artery disease (CAD) with normal baseline wall motion.

**Methods:**

Eighty-nine patients who referred for coronary angiography due to suspected of CAD were included. Forty patients with severe coronary artery stenosis were diagnosed with significant CAD, and 49 were defined as non-significant CAD. Stress echocardiography was performed 24 h before angiography. Layer-specific longitudinal strains were assessed from the endocardium, mid-myocardium, and epicardium by 2D speckle-tracking echocardiography. Binary logistic regression analyses were performed to evaluate the association between significant CAD and echocardiographic parameters. A receiver operating characteristic curve was used to assess the capability of layer-specific strain and GWE to diagnose significant CAD.

**Results:**

Patients with significant CAD had the worse function in all three myocardial layers at peak exercise compared with those with non-significant CAD when assessed with global longitudinal strain (GLS). At the peak exercise and recovery periods, GWE was lower in patients with significant CAD than in patients with non-significant CAD. In multivariable binary logistic regression analysis, peak endocardial GLS (OR: 1.35, *p* = 0.006) and peak GWE (OR: 0.76, *p* = 0.001) were associated with significant CAD. Receiver operating characteristic curves showed peak GWE to be superior to mid-myocardial, epicardial, and endocardial GLS in identifying significant CAD. Further, adding peak GWE to endocardial GLS could improve diagnostic capabilities.

**Conclusions:**

Both GWE and endocardial GLS contribute to improving the diagnostic performance of exercise stress echocardiography. Furthermore, adding peak GWE to peak endocardial GLS provides incremental diagnostic value during a non-invasive screening of significant CAD before radioactive or invasive examinations.

## Introduction

Coronary artery disease (CAD) is one of the leading global causes of mortality and morbidity. Concerning the diagnostics of CAD, coronary angiography (CAG), which despite being considered the gold standard, only confirmed significant CAD in 38% of suspected patients (1). Stress echocardiography, which is recommended as a first-line diagnostic test in patients with suspected of CAD, showed a limited specificity and sensitivity due to lacking quantitative and objective methods (2). Non-invasive, quantitative, and objective imaging techniques are necessary for an optimal decision on diagnosis and therapy in patients suspected of CAD without known heart disease to improve clinical outcomes and enhance the diagnostic yield of cardiac catheterization.

The newly developed and explored (3–5) quantitatively objective method of layer-specific strain evaluation could increase the diagnostic accuracy for CAD (5) by evaluating the endocardial myocardium's longitudinal function, which was more susceptible to ischemic injury.

However, as strain is load-dependent, increasing the afterload may underestimate the left ventricular (LV) function; therefore, myocardial work (MW), which combines strain and non-invasive LV pressure, could overcome the limitation. Global myocardial work efficiency (GWE) is one of the major MW parameters and is derived from the percentage ratio of constructive work to the sum of constructive work and wasted work. Recently, significantly lower GWE values were reported in patients with CAD, heart failure, hypertension with left ventricle hypertrophy, and COVID-19 (6–10), and the prognostic value of GWE was found to predict the long-term outcomes of patients after ST-segment elevation myocardial infarction (11) or patients after cardiac resynchronization therapy (12).

Due to the limited data available, this study aims to compare the diagnostic value of GWE with myocardial layer-specific global longitudinal strain during exercise stress to detect significant CAD with normal baseline wall motion.

## Materials and Methods

### Study Population

The study was conducted in a single tertiary coronary care center with 89 patients without known ischemic heart disease who were referred with angina pectoris and had plans to undergo treadmill exercise stress testing and CAG. Patients with the following criteria were excluded: age <18 years; baseline LV ejection fraction <50%; abnormal baseline wall motion; previous myocardial infarction; prior coronary artery bypass grafting or coronary interventional therapy; left bundle branch block; atrial fibrillation; sustained severe arrhythmia; severe valvular dysfunction; inability to undergo exercise testing; absence of any of apical four-chamber, three-chamber, and two-chamber views; and inadequate image quality. All patients underwent two-dimensional (2D) echocardiography (speckle-tracking echocardiography and MW analysis), and a treadmill exercise stress test followed by CAG.

Written informed consent was given by all study participants or their legal representatives. The study complied with the Declaration of Helsinki and was approved by the Ethics Committees of Fuwai Hospital (no. 2018-1121).

### Conventional Echocardiography

Comprehensive conventional echocardiography was performed (13) using a commercially available ultrasound system (Vivid E9 or Vivid E95, GE Healthcare, Horton, Norway) with a 3.5-MHz transducer. Images were analyzed offline using dedicated software (EchoPAC 203, GE Healthcare, Horton, Norway). LV dimensions, LV septal thickness, and LV posterior wall thickness were measured from the parasternal long-axis view. LV mass was calculated as {0.8 × 1.04 × [(LV end-diastolic diameter + LV end-diastolic posterior wall thickness + end-diastolic septal wall thickness)^3^ – (LV end-diastolic diameter)^3^] + 0.6}. LV mass was then indexed for body surface area to generate the LV mass index. LV ejection fraction was measured using Simpson's biplane method from apical four- and two-chamber views. Early trans-mitral velocity (E wave) and late trans-mitral velocity (A wave) were measured by pulsed-wave Doppler from the apical four-chamber view with the sample volume positioned at the tip of the mitral leaflets. Mitral inflow E/A ratio was calculated as E wave divided by A wave.

### Treadmill Exercise Stress Echocardiography

The treadmill exercise stress test was conducted utilizing the standard Bruce protocol (14) whereby the patient's heart rate, blood pressure, and 12-lead electrocardiography (ECG) were recorded. Cine loops (2D) from the four-, two-, and three-chamber apical views were taken during the rest (before exercise), peak exercise (<1 min after exercise), and recovery (3 min after exercise) periods. Criteria for terminating the test were achieving a target heart rate of 85% of the age-predicted maximum, development of wall motion abnormality, development of intolerant symptoms, severe ischemic electrocardiographic changes, severe hypertension (systolic blood pressure >220 mmHg or diastolic blood pressure >120 mmHg), symptomatic hypotension, or significant arrhythmia (14). The ECGs were categorized as either normal or abnormal by two blinded investigators. A positive exercise ECG was defined as ST-segment horizontal or down-sloping depression ≥1 mm with duration >2 min or ST-segment elevation ≥1 mm with duration >1 min in the leads dominated by R waves. The regional myocardial functional abnormality was assessed based on the observed wall thickening and endocardial motion of the myocardial segment in exercise stress echocardiography (15). A 17-segment model was used to assess wall motion from the apical four-, two-, and three-chamber views during the rest, peak exertion, and recovery periods, and a semiquantitative scoring system was used to analyze each segment (1 = normal wall motion, 2= hypokinesia, 3 = akinesia, and 4 = dyskinesia) (15, 16). The wall motion score index (WMSI) was calculated for each patient as the average of the analyzed segmental values, and patients with wall motion abnormality (WMSI > 1) during exercise were considered as positive stress echocardiogram.

### Speckle-Tracking Echocardiography and Myocardial Layer-Specific Strain

**Figure 1:**
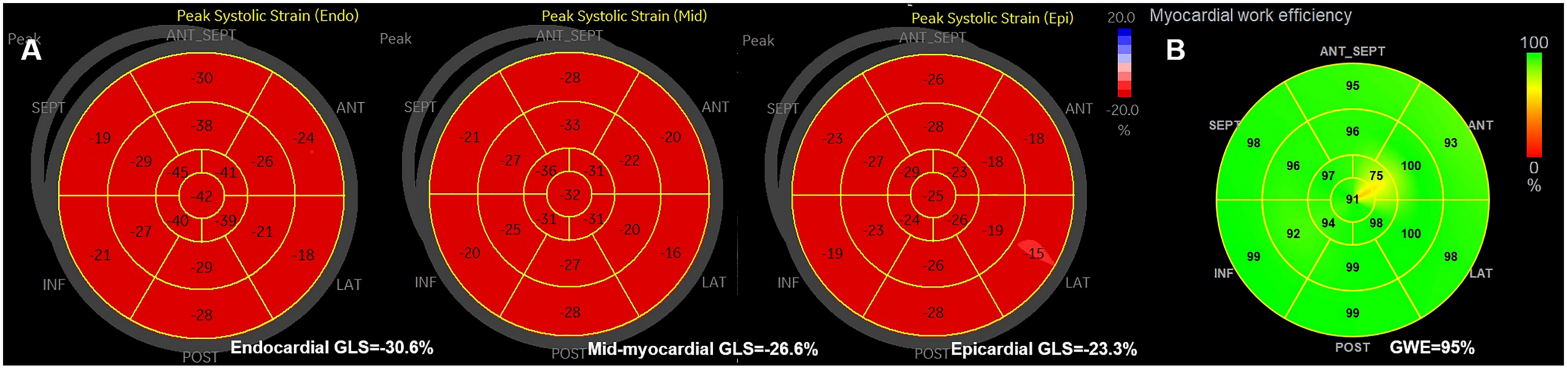
**(A)** The example of bull's eyes of the global myocardial longitudinal layer-specific strain. **(B)** The example of global myocardial work efficiency. GLS, global longitudinal strain; GWE, global myocardial work efficiency.

From each of the three apical views at rest and during exercise, the cardiac loop with the best representation of the LV wall was identified and analyzed in the commercially available software (EchoPAC version 203, GE Healthcare); initially with automated function imaging (AFI) and subsequently with the 2D strain software. AFI identified and tracked the endocardium automatically to generate the LV global longitudinal strain (GLS), and the regional speckle area of interest was adjusted manually where the tracking was poor. For 2D strain analysis, the endocardial borders were manually delineated by the operator and then traced by the software in the end-systolic frame for the analyses of longitudinal endocardial, mid-myocardial, and epicardial strains (Figure 1). Images with low tracking quality in more than two segments in a single view were excluded from further analysis. The Δ-value of the LV myocardial layer-specific measurements was calculated as the difference between the values at peak exercise stress and at rest.

### Global LV Myocardial Work Efficiency Analysis

Global myocardial work efficiency was calculated from a combination of the 2D LV GLS (based on AFI) and a non-invasively estimated LV pressure, using the commercially obtained software (EchoPAC version 203, GE Healthcare) (17). Brachial blood pressure was measured immediately after the echocardiography test, as a surrogate of non-invasive peak LV pressure. A non-invasive pressure-strain loop was constructed from the software adjusted to the timing of valvular events, mitral valve closure, aortic valve opening, aortic valve closure, and mitral valve opening (18). Myocardial work index corresponds to the area of the pressure-strain loop during the cardiac cycle. Myocardial constructive work was defined as work performed by the myocardium during segmental shortening in systole or during lengthening in isovolumic relaxation. Myocardial wasted work was defined as work performed by the myocardium during segmental lengthening in systole or during segmental shortening against a closed aortic valve in isovolumic relaxation. GWE was calculated as the sum of constructive work in all LV segments, divided by the sum of constructive and wasted work in all LV segments, expressed as a percentage (Figure 1).

### Coronary Angiography

The images from CAG were interpreted visually by an experienced cardiologist blinded to patients' echocardiographic data. Significant CAD was defined as ≥70% luminal diameter narrowing in one or more major epicardial vessels or ≥50% in the left main coronary (19).

### Intra- and Interobserver Variability for Myocardial Layer-Specific Strain and GWE

Intra- and interobserver variabilities were determined with 15 randomly selected patients, where the analysis was repeated by the same investigator 2 weeks later and by a second investigator blinded to the initial results of the first investigator, respectively.

### Statistical Analysis

Normally distributed continuous variables are presented as mean values ± standard deviation or as the median value (interquartile range) when not normally distributed. Categorical variables are presented as absolute numbers and percentages. Normal distribution was verified by the Shapiro–Wilk test. Continuous variables were compared using Student's *t*-test or Mann–Whitney *U*-test, as appropriate, whereas categorical variables were compared with the chi-squared test or Fisher's exact test. Correlation between continuous variables was performed using Pearson's or Spearman's correlation coefficient. Univariate binary logistic regression analyses were performed to evaluate the association between the presence of significant CAD and the echocardiographic variables. After excluding variables that showed collinearity (Pearson's or Spearman's correlation coefficient ≥ 0.60 or variance inflation factor > 10), all variables that found to be significant on univariate binary logistic regression were considered for multiple binary regression analysis using the forward selection method. Receiver operating characteristic curve analysis and Youden's index were used to assess the optimal cutoff points for layer-specific strain and GWE in predicting significant CAD. The comparisons of AUCs were performed using the method described by DeLong et al. (20). Intraclass correlation coefficients and coefficients of variation were calculated during the inter- and intraobserver variability tests to evaluate the reproducibility.

Analyses were performed using SPSS, version 25.0 (IBM Corp., Armonk, NJ, USA), MedCalc, version 18.2.1 (MedCalc Software, Ltd., Ostend, Belgium), and GraphPad Prism 8 (GraphPad Software, San Diego, CA, USA). All statistical tests were two-sided, and a *p* < 0.05 was considered statistically significant.

## Results

### Clinical Characteristics

Of the 89 patients, 40 and 49 patients were diagnosed with significant and non-significant CAD by CAG. The demographic information, angiographic results, and exercise ECG results of both groups are presented in Table 1, and hemodynamic data during treadmill exercise testing are shown in Table 2. No statistically significant differences were observed (all *p* > 0.05) between the two groups regarding age, gender, heart rate, blood pressure, coronary dominance, and exercise ECG results. Patients with significant CAD showed a larger body surface area and body mass index (both *p* < 0.05) and a higher incidence of dyslipidemia (*p* < 0.05) compared with those in the non-significant CAD group (Table 1).

**Table 1:** Clinical characteristics and conventional echocardiographic variables of patients.

**Variable**	**Non-Significant CAD (*n* = 49)**	**Significant CAD (*n* = 40)**	***p*-value**
Age, years	56 ± 8	58 ± 8	0.202
Male, *n* (%)	30 (61)	31 (78)	0.100
Body surface area, m^2^	1.79 ± 0.17	1.87 ± 0.18	0.033
Body mass index, kg/m^2^	25.3 ± 3.1	27.4 ± 3.1	0.002
**Medical history**, ***n*** **(%)**			
Hypertension	23 (47)	27 (68)	0.052
Diabetes mellitus	14 (29)	14 (35)	0.516
Dyslipidemia	40 (82)	39 (98)	0.043
**Medication**, ***n*** **(%)**			
Platelet inhibitors	34 (69)	37 (93)	0.007
Beta-Blockers	25 (51)	33 (83)	0.002
Calcium channel blockers	22 (45)	15 (38)	0.481
ACE inhibitors or ARBs	11 (22)	20 (50)	0.007
Statins	42 (86)	38 (95)	0.275
**Coronary angiography**, ***n*** **(%)**			
One-Vessel disease	–	27 (68)	–
Two-Vessel disease	–	9 (23)	–
Three-Vessel disease	–	4 (10)	–
Left main coronary artery	–	2 (5)	–
Left anterior descending artery	–	29 (73)	–
Left circumflex artery	–	10 (25)	–
Right coronary artery	–	16 (40)	–
**Coronary dominance**, ***n*** **(%)**			
Right coronary dominance	45 (92)	36 (90)	>0.999
Left coronary dominance	3 (6)	3 (8)	>0.999
Codominance	1 (2)	1 (3)	>0.999
ECG positive, *n* (%)	15 (31)	7 (18)	0.154
**Echocardiographic parameters**			
LVDd, mm	47 ± 3	48 ± 5	0.239
LVDs, mm	32 ± 3	31 ± 3	0.325
IVSd, mm	9 ± 1	9 ± 1	0.338
LVPWd, mm	8 ± 1	9 ± 1	0.016
LV mass index, g/m^2^	78 ± 16	82 ± 15	0.173
Mitral E, m/s	0.8 ± 0.2	0.8 ± 0.2	0.509
Mitral A, m/s	0.8 ± 0.2	0.8 ± 0.1	0.531
Mitral E/A	1.0 ± 0.3	1.0 ± 0.3	0.683

*ACE, angiotensin-converting enzyme; ARB, angiotensin II receptor blocker; CAD, coronary artery disease; ECG, electrocardiography; LV, left ventricular; LVDd, LV end-diastolic diameter; LVDs, LV end-systolic diameter; LVPWd, LV posterior wall thickness in end-diastole; Mitral E, mitral early-diastole velocity; Mitral A, mitral late-diastole velocity; IVSd, interventricular septum thickness in end-diastole*.

*Data are expressed as mean ± SD or as number (percentage)*.

**Table 2:** Hemodynamic and echocardiographic parameters at rest and during treadmill exercise stress.

**Variable**	**Non-Significant CAD (*n* = 49)**	**Significant CAD (*n* = 40)**	***P*-Value**
**Rest**			
Heat rate, beats/min	77 ± 15	75 ± 13	0.523
SBP, mm Hg	132 ± 20	131 ± 18	0.914
DBP, mm Hg	78 ± 12	79 ± 12	0.581
LVEF, %	64 ± 5	62 ± 4	0.203
Rest GLS(AFI), %	−19.6 ± 2.3	−19.2 ± 1.8	0.323
Rest endocardial GLS, %	−24.0 ± 3.0	−23.9 ± 2.6	0.867
Rest mid–myocardial GLS, %	−21.0 ± 2.9	−20.9 ± 2.3	0.853
Rest epicardial GLS, %	−18.5 ± 2.8	−18.4 ± 2.1	0.825
Rest GWE, %	95 (93, 96)	94 (92, 96)	0.060
**Peak exercise**			
Heat rate, beats/min	140 ± 8	138 ± 7	0.161
SBP, mmHg	176 ± 20	171 ± 22	0.268
DBP, mmHg	77 ± 14	77 ± 15	0.966
LVEF, %	67 ± 3	66 ± 4	0.180
Peak WMSI	1.00 (1.00, 1.00)	1.00 (1.00, 1.06)	0.079
Peak GLS(AFI), %	−21.5 ± 2.3	−20.0 ± 1.9	0.001
Peak endocardial GLS, %	−28.8 ± 2.7	−26.0 ± 3.0	<0.001
Peak mid-myocardial GLS, %	−25.0 ± 2.4	−22.8 ± 2.7	<0.001
Peak epicardial GLS, %	−21.8 ± 2.3	−19.9 ± 2.4	<0.001
ΔEndocardial GLS, %	−4.86 ± 3.00	−2.17 ± 3.49	<0.001
ΔMid-myocardial GLS, %	−4.02 ± 2.72	−1.90 ± 3.12	0.001
ΔEpicardial GLS, %	−3.30 ± 2.67	−1.57 ± 2.84	0.004
Peak GWE, %	94 (93, 95)	90 (87, 93)	<0.001
**Recovery**			
Heat rate, beats/min	84 ± 12	83 ± 10	0.564
SBP, mmHg	155 ± 22	159 ± 26	0.409
DBP, mmHg	69 ± 11	72 ± 16	0.377
LVEF, %	67 ± 5	66 ± 6	0.354
Recovery WMSI	1.00 (1.00, 1.03)	1.00 (1.00, 1.06)	0.702
Recovery GLS(AFI), %	−21.4 ± 2.7	−20.7 ± 2.0	0.195
Recovery endocardial GLS, %	−27.0 ± 3.5	−26.9 ± 2.8	0.855
Recovery mid-myocardial GLS, %	−23.4 ± 3.0	−23.6 ± 2.6	0.841
Recovery epicardial GLS, %	−20.4 ± 2.5	−20.8 ± 2.4	0.562
Recovery GWE, %	95 (94, 96)	93 (91, 95)	0.001

*AFI, Automated function imaging; CAD, coronary artery disease; DBP, diastolic blood pressure; EF, ejection fraction; GLS, global longitudinal strain; GWE, global myocardial work efficiency; LV, left ventricular; SBP, systolic blood pressure; WMSI, wall motion score index*.

*Δ-value, stress value—rest value. Data are expressed as mean ± SD or as median (interquartile range)*.

### Conventional Echocardiography

No significant differences were observed (all *p* > 0.05) between the two groups for the conventional echocardiographic parameters of LV end-diastolic or end-systolic dimensions, LV septal thickness in end-diastole, LV mass index, mitral early-diastole velocity, mitral late-diastole velocity, and the ratio of mitral early-diastole velocity to late-diastole velocity. LV posterior wall thickness in end-diastole was significantly larger in the significant CAD group than that of the non-significant CAD group (*p* < 0.05; Table 1).

### GLS and Myocardial Layer-Specific Strain Analysis

The LV GLS (AFI) had significantly lower absolute values during peak exercise in the significant CAD group (*p* = 0.001) compared with those in the non-significant group, although there were no significant differences observed during the rest or recovery periods **(**all *p* > 0.05; Table 2).

**Figure 2:**
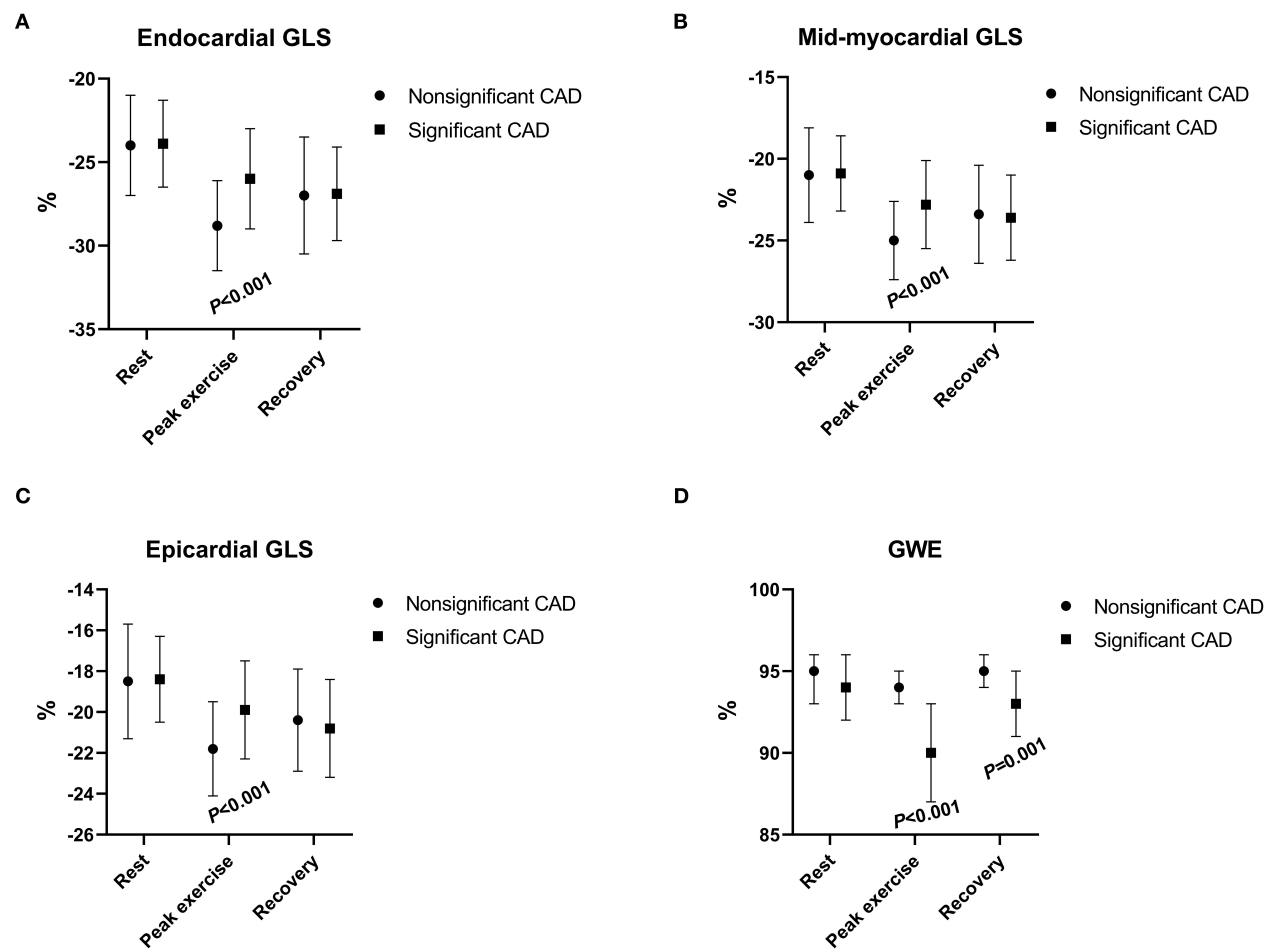
The changes in myocardial longitudinal layer-specific strain [**(A)** endocardial GLS; **(B)** mid-myocardial GLS; **(C)** and epicardial GLS] and global myocardial work efficiency **(D)** between patients with nonsignificant CAD and significant CAD during rest, peak exercise, and recovery period. CAD, coronary artery disease; GLS, global longitudinal strain; GWE, global myocardial work efficiency.

Patients with significant CAD had a significantly worse function than those with non-significant CAD at peak exercise in all three myocardial layers when assessed with GLS (all *p* < 0.001), although the differences between the two groups were not significant at rest or during recovery (*p* > 0.05; Figure 2, Table 2). The difference (Δ-value) in endocardial, mid-myocardial, and epicardial GLS from rest to peak exercise were, respectively significantly smaller in the significant CAD group than that of patients with non-significant CAD (all *p* < 0.01; Table 2).

### Global LV Myocardial Work Efficiency Analysis

The GWE value was minimal during peak exercise and increased in the recovery period (Figure 2) in both groups. At peak exercise and recovery period, the values of GWE in patients with significant CAD were significantly lower than those of the non-significant CAD group (all *p* < 0.01), although at rest, the values of the two groups had no significant differences (*p* > 0.05; Table 2).

### Correlation Between GWE and Myocardial Strain at Peak Exercise

Peak GWE showed modest negative correlations with peak endocardial, mid-myocardial, and epicardial GLS (*r* = −0.44, −0.43, and −0.43, respectively; all *p* < 0.001) and a good negative correlation with peak GLS using AFI (*r* = −0.63, *p* < 0.001; Supplementary Figure 1). Peak endocardial, mid-myocardial, and epicardial GLS (*r* = 0.46, 0.47, and 0.45, respectively, all *p* < 0.001) all presented modest positive correlations with peak GLS using AFI (Supplementary Figure 2). Peak endocardial GLS showed great correlations with both peak mid-myocardial GLS and peak epicardial GLS (*r* = 0.98, and 0.93, respectively, both *p* < 0.001).

### Binary Logistic Regression Analyses for the Detection of Significant CAD

In univariable binary logistic regression analysis, body surface area, body mass index, dyslipidemia, LV posterior wall thickness in end-diastole, peak endocardial, mid-myocardial, epicardial GLS, peak GLS (AFI), peak GWE, and recovery GWE were significantly associated with significant CAD (Table 3).

**Table 3:** Univariate and multivariate binary logistic regression analyses for detection of significant CAD.

**Parameters**	**Univariate binary logistic regression**		**Multivariate binary logistic regression**	
	**OR [95% CI]**	***p*-value**	**OR [95% CI]**	***p*-value**
Body surface area, m^2^	14.97 [1.19–189.02]	0.036		
Body mass index, kg/m^2^	1.24 [1.07–1.44]	0.004		
Dyslipidemia	8.78 [1.06–72.56]	0.044		
LVPWd, mm	1.57 [1.07–2.30]	0.021		
Peak endocardial GLS, %	1.44 [1.19–1.75]	<0.001	1.35 [1.09–1.67]	0.006
Peak mid-myocardial GLS, %	1.44 [1.18–1.78]	0.001		
Peak epicardial GLS, %	1.43 [1.15–1.77]	0.001		
Peak GLS(AFI), %	1.39 [1.13–1.72]	0.002		
Peak GWE, %	0.71 [0.60–0.84]	<0.001	0.76 [0.64–0.90]	0.001
Recovery GWE, %	0.81 [0.68–0.96]	0.013		

*CI, confidence interval; OR, odds ratio; for other abbreviations see Tables 1, 2*.

In multivariable binary logistic regression analysis, peak endocardial GLS (odds ratio: 1.35, *p* = 0.006) and peak GWE (odds ratio: 0.76, *p* < 0.001) were significantly associated with significant CAD (Table 3).

### Receiver Operating Characteristic Curve Analysis for the Detection of Significant CAD

**Figure 3:**
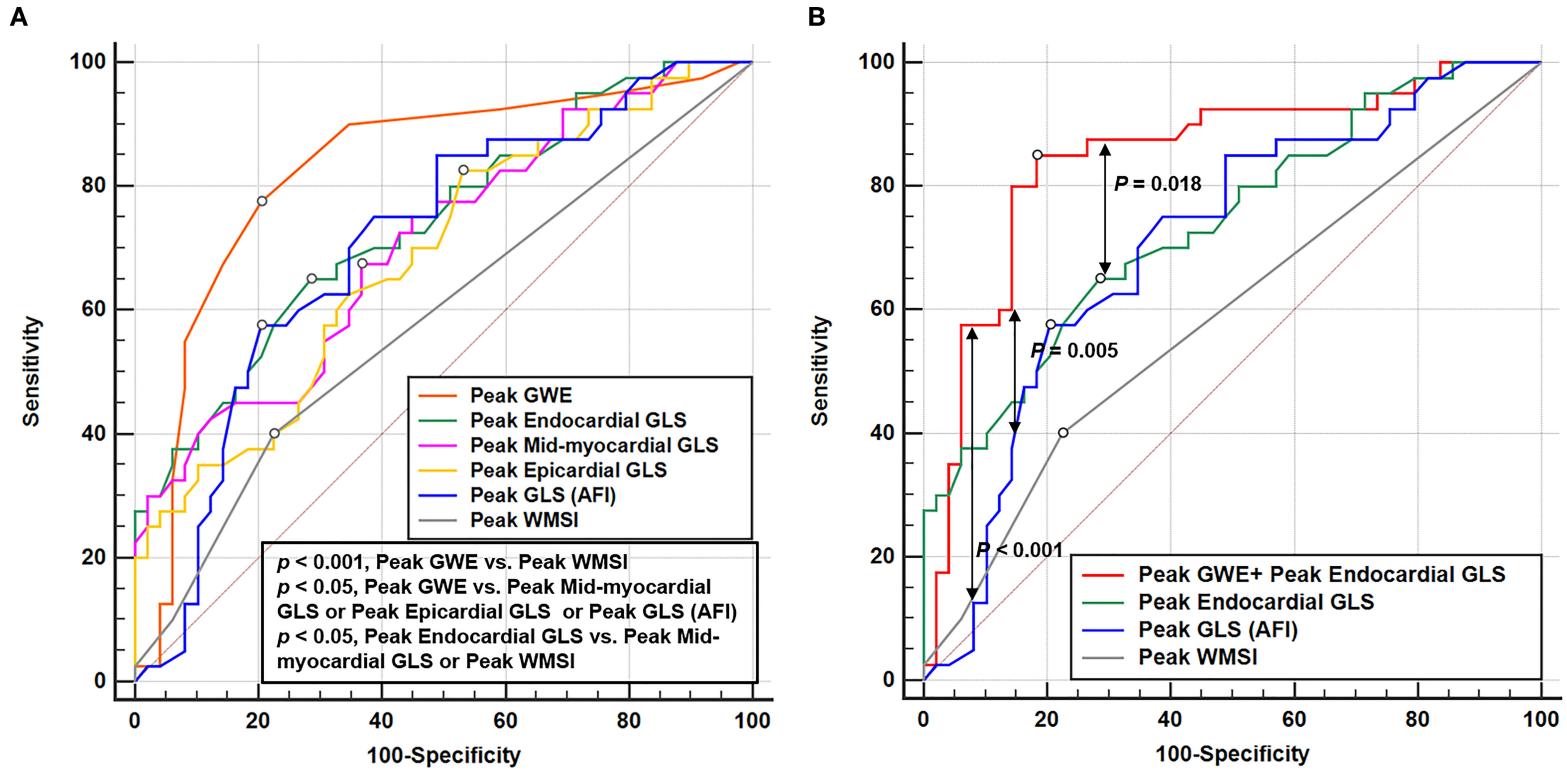
Receiver operating characteristic curves of echocardiographic parameters at peak exercise to detect the significant CAD. **(A)** Receiver operating characteristic curves of GWE, layer-specific strain, GLS (AFI), and WMSI at peak exercise in identifying significant CAD. **(B)** Receiver operating characteristic curve of the parameter combined peak GWE and peak endocardial GLS, peak endocardial GLS, peak GLS (AFI), and peak WMSI in detecting significant CAD. CAD, coronary artery disease; GLS, global longitudinal strain; GWE, global myocardial work efficiency; AFI, automated function imaging; WMSI, wall motion score index.

According to receiver operating characteristic curve analysis, the cutoff value for the best possible detection of peak GWE in the significant CAD group was 92%. Peak GWE had the highest area under the curve (AUC) for the detection of significant CAD (AUC, 0.827; *p* < 0.001) that was superior to peak mid-myocardial and epicardial GLS (AUC, 0.708, and 0.688, respectively, both *p* < 0.05; Figure 3A, Table 4), although it was not statistically better than endocardial GLS (AUC: 0.739, *p* > 0.05). However, the addition of peak GWE to peak endocardial GLS significantly increased the AUC over that of peak endocardial GLS (AUC: 0.848 vs. 0.739, *p* = 0.018; Figure 3B, Table 4). The AUCs of the combination of peak GWE and peak endocardial GLS or peak GWE alone were significantly higher than that of peak GLS using AFI (Figures 3A,B, Table 4). The AUC of peak GLS (AFI) was similar to those of peak myocardial layer-specific GLS (0.708 vs. endocardial, 0.739; mid-myocardial, 0.708, and epicardial, 0.688; all *p* > 0.05; Figure 3A, Table 4). The AUCs of peak myocardial layer-specific GLS and peak GWE were better than peak WMSI, which represented the conventional exercise stress echocardiography (Figure 3A, Table 4). The Δ-value of myocardial layer-specific GLS also had high AUCs to identify significant CAD (endocardial, 0.721; mid-myocardial, 0.702; and epicardial, 0.684; Table 4).

**Table 4:** Receiver operating characteristic curve analysis for the detection of significant CAD.

**Parameters**	**AUC**	**95%CI**	***p*-value**	**Cutoff value**	**Sensitivity (%)**	**Specificity (%)**	**Youden's index**
Peak GWE + Peak endocardial GLS	0.848	0.756–0.915	<0.001	>0.406	85.0	81.6	0.666
Peak GWE, %	0.827	0.733–0.899	<0.001	≤ 92	77.5	79.6	0.571
Peak endocardial GLS, %	0.739	0.635–0.827	<0.001	>-27.0	65.0	71.4	0.364
Peak mid-myocardial GLS, %	0.708	0.602–0.800	<0.001	>-24.0	67.5	63.3	0.308
Peak epicardial GLS, %	0.688	0.581–0.782	0.001	>-21.8	82.5	46.9	0.294
Peak GLS (AFI), %	0.708	0.603–0.800	<0.001	>-20.0	57.5	79.6	0.371
Peak WMSI	0.587	0.478–0.691	0.080	>1.00	40.0	77.6	0.176
ΔEndocardial GLS, %	0.721	0.616–0.811	<0.001	>-3.6	67.5	71.4	0.389
ΔMid-myocardial GLS, %	0.702	0.596–0.795	<0.001	>-1.5	50.0	85.7	0.357
ΔEpicardial GLS, %	0.684	0.577–0.778	0.001	>-4.4	85.0	49.0	0.340

*AUC, area under the curve; CI, confidence interval; Δ-value, stress value—rest value; for other abbreviations see as Tables 1, 2*.

### Intra- and Interobserver Variabilities for Layer-Specific Strain Parameters and GWE

The intra- and interobserver variabilities for peak myocardial layer-specific GLS and peak GWE, which showed good repeatability and reproducibility, are summarized in Supplementary Table 1.

## Discussion

This is the first study to compare the ability of myocardial layer-specific strain and GWE in identifying significant CAD in patients with angina pectoris without a known history of CAD. The primary findings were that both myocardial layer-specific GLS and GWE at peak exercise can discriminate significant CAD. At peak exercise, GWE was superior to myocardial layer-specific GLS as a non-invasive measure for the detection of significant CAD. Furthermore, the combination of peak GWE and peak endocardial GLS had incremental diagnostic value for the detection of significant CAD when compared to that of myocardial layer-specific GLS at peak exercise alone.

### Myocardial Layer-Specific Strain for the Detection of Significant CAD Compared With GLS Based on AFI or WMSI During Exercise Stress

Previous studies have reported excellent intra- and interobserver reproducibilities of layer-specific strain (21, 22). In addition, studies have also investigated the diagnostic value of layer-specific strain on stress echocardiography in patients suspected of CAD (23–26). Nishi et al. suggested that layer-specific GLS at the early recovery phase after cycle ergometer exercise stress was significantly more impaired in the ischemic territories than in non-ischemic territories (26). However, in our research, the differences in layer-specific strain during the rest or recovery periods between significant and non-significant CAD groups were less pronounced. Only layer-specific strain at peak exercise after treadmill exercise stress testing provided a high value for the non-invasive identification of significant CAD. Despite the statement of the stress period is different, the definition of the early recovery period in the study of Nishi et al. was similar to the definition of peak exercise in our research (from immediate cessation to <3 min after exercise vs. <1 min after treadmill exercise), and the definition of the recovery period in our research was >3 min after treadmill exercise. Therefore, the results are consistent.

The LV heart wall comprises three layers: the oblique endocardial, the circular mid-myocardial, and the oblique epicardial layer. Since the endocardial layer of the myocardium is more susceptible to ischemia than the epicardial layer, and the endocardial-layer fibers are mainly oriented in the longitudinal direction (27, 28), it is likely to expect in patients with CAD that ischemia extends from endocardium to epicardium and endocardial GLS deteriorates before epicardial GLS abnormalities become apparent (29). However, the previous studies did not demonstrate clear superiority for endocardial-layer strain over other layers on stress echocardiography for the detection of significant CAD (23, 26). Nishi et al. found that the diagnostic ability of the endocardial, mid-myocardial, and epicardial GLS at the early recovery phase on cycle ergometer exercise stress echocardiography (AUC; 0.621, 0.619, and 0.616, respectively), are comparable (26), which was also found by Ejlersen et al. (23). Similarly, in this study, the myocardial layer-specific GLS analysis revealed that all myocardial layers were affected in patients with significant CAD at peak exercise. However, the AUCs for the detection of significant CAD were not statistically different among the three layers at peak exercise although the AUC of endocardial GLS was the highest among all three layers.

In line with previous research (23, 26, 30), myocardial layer-specific GLS was better than the semiquantitative method of WMSI in stress echocardiography to identify significant CAD. Besides, with previous studies demonstrated that GLS added value to exercise stress testing to diagnose various cardiac diseases, including myocardial ischemia (30, 31). We also compared the diagnostic value of layer-specific GLS and GLS (AFI) during exercise stress to identify significant CAD and found that myocardial layer-specific GLS was relative to GLS (AFI). However, no statistical differences were observed in the diagnostic value of the three layer-specific GLS and GLS (AFI) when applied to treadmill exercise stress echocardiography, although the AUC of endocardial GLS was better than that of GLS (AFI), which were consistent with previous studies (23, 25). Therefore, it was confirmed that endocardial GLS at peak exercise was superior to the conventional stress echocardiography and has no significant superiority over GLS (AFI) or other layer-specific strain in identifying myocardial ischemia.

In addition, we also explored the difference in endocardial, mid-myocardial, and epicardial GLS from rest to peak exercise and found that significant CAD can be effectively identified. The diagnostic value of the differences in endocardial, mid-myocardial, and epicardial GLS from rest to peak exercise was comparable to endocardial, mid-myocardial, and epicardial GLS at peak exercise.

### Incremental Diagnostic Value of GWE

The myocardial layer-specific GLS had a high diagnostic value for significant CAD, but these parameters were still limited by load dependency. An increase in afterload would lead to a reduction in strain, which may underestimate the true myocardial contractility, especially in the context of stress echocardiography (32). MW can overcome this limitation and reflect the true contractility of the myocardium by establishing the GWE, which combines the global constructive and wasted work of the myocardium, which can better reflect the myocardial changes affected by ischemia.

Recently, various studies have proved the diagnostic and prognostic values of GWE (6–12). However, the comparison of the diagnostic capability of GWE and myocardial layer-specific GLS during peak exercise in identifying significant CAD in patients without a history of CAD has not been explored prior to our study. We observed that the GWE was moderately correlated with the myocardial layer-specific GLS at peak exercise but stronger with the GLS obtained by AFI, because MW derived from GLS (based on AFI) and brachial artery blood pressure. The peak GWE was superior in the detection of significant CAD when compared to that of the WMSI, GLS (AFI), mid-myocardial, and epicardial GLS at peak. However, the diagnostic capability of peak GWE did not show statistically significant superiority over peak endocardial GLS, although the AUC of peak GWE was higher than that of peak endocardial GLS. The combination of peak GWE and peak endocardial GLS could significantly improve the discrimination ability for significant CAD when compared to endocardial GLS alone. The present research extends the usefulness of peak GWE to patients with CAD with normal LV ejection fraction and baseline wall motion and suggests an incremental diagnostic value of peak GWE over the myocardial layer-specific GLS in patients whose rest echocardiography does not suggest CAD. The MW can only indicate overall-layer myocardial function but could not reflect each layer's MW at present, which can be explored in future studies.

### Clinical Implications

In patients with angina pectoris and no history of CAD, conventional echocardiography during exercise stress was not accurate enough to detect significant CAD. The use of GWE or myocardial layer-specific GLS at peak exercise, especially the combination of peak GWE and peak endocardial GLS, provided a better non-invasive screening method to identify significant CAD before radioactive or invasive exams. Peak GWE is particularly important in measurements performed during exercise stress testing, or in patients with inadequate blood pressure control. However, the practical applicability of these new parameters to assist the decision-making process for significant CAD needs to be verified in further studies.

## Limitations

This was a retrospective, single-center study, and prospective validation is needed. Selection bias inherent in any study comparing stress echocardiography with invasive angiography may have occurred in our research since only patients with chest pain who underwent invasive angiography were included. The circumferential and radial strains were not investigated in this study, since the myocardial fibers most vulnerable to ischemia are the longitudinally orientated fibers that are located subendocardial, and longitudinal strain is thought to be the most sensitive parameter to detect CAD (33). Since LV GWE is predicated on the measurement of GLS, it is not a vendor-independent measure and is still influenced by the image quality. Fractional flow reserve measurements were not taken in this study, and therefore, the true hemodynamic relevance of the stenoses is not known. Despite this limitation, the fraction of patients with non-flow limiting stenosis may have been reduced since a high cutoff for significant CAD was used.

## Conclusion

In patients with angina pectoris and normal baseline wall motion, both peak GWE and peak endocardial GLS are independent predictors of significant CAD that can significantly improve the diagnostic performance of exercise stress echocardiography. Furthermore, adding peak GWE to peak endocardial GLS provides incremental diagnostic value in non-invasive screening for significant CAD before radioactive or invasive examinations.

## Data Availability Statement

The raw data supporting the conclusions of this article will be made available by the authors, without undue reservation.

## Ethics Statement

The studies involving human participants were reviewed and approved by Ethics Committees of Fuwai Hospital (No. 2018-1121). The participants or their legal representatives provided their written informed consent to participate in this study.

## Author Contributions

WW, ZZ, and HW contributed to the conception, design, supervision of the study, and revised the manuscript critically for intellectual content. JL drafted the manuscript. LG, JH, LN, YZ, XL, and JW contributed to the collection and interpretation of data. JL, ML, and YC contributed to statistical analysis. All authors contributed to the article and approved the submitted version.

## Funding

This study was supported by the Beijing Municipal Science and Technology Commission (Grant No. Z171100001017213) and Construction Research Project of Key Laboratory (Cultivation) of Chinese Academy of Medical Sciences (Grant No. 2019PT310025).

## Conflict of Interest

The authors declare that the research was conducted in the absence of any commercial or financial relationships that could be construed as a potential conflict of interest.

## Publisher's Note

All claims expressed in this article are solely those of the authors and do not necessarily represent those of their affiliated organizations, or those of the publisher, the editors and the reviewers. Any product that may be evaluated in this article, or claim that may be made by its manufacturer, is not guaranteed or endorsed by the publisher.

## Supplementary Material

Supplementary Figure 1 Correlation between GWE and layer-specific strain, GLS (AFI) at peak exercise. **(A)** Correlation between peak GWE and peak endocardial GLS. **(B)** Correlation between peak GWE and peak mid-myocardial GLS. **(C)** Correlation between peak GWE and peak epicardial GLS. **(D)** Correlation between peak GWE and peak GLS (AFI). GLS, global longitudinal strain; GWE, global myocardial work efficiency; AFI, automated function imaging.

Supplementary Figure 2 Correlation between layer-specific strain and GLS (AFI) at peak exercise. **(A)** Correlation between peak endocardial GLS and peak GLS (AFI). **(B)** Correlation between peak mid-myocardial GLS and peak GLS (AFI). **(C)** Correlation between peak epicardial GLS and peak GLS (AFI). GLS, global longitudinal strain; AFI, automated function imaging.

Supplementary Table 1 Intra- and inter-observer variabilities for peak layer-specific strain parameters and peak GWE. ICC, Intraclass correlation coefficient; CI, confidence interval; CV, coefficient of variation; GLS, global longitudinal strain; GWE, global myocardial work efficiency.

## Abbreviations

CAD: coronary artery disease
2D: two-dimensional
MW: myocardial work
LV: left ventricular
GWE: global myocardial work efficiency
GLS: global longitudinal strain
CAG: coronary angiography
ECG: electrocardiography
WMSI: wall motion score index
AFI: automated function imaging
AUC: area under the curve.
