# Supplementary figures and images for: Comparison of Myocardial Layer-Specific Strain and Global Myocardial Work Efficiency During Treadmill Exercise Stress in Detecting Significant Coronary Artery Disease
Source: Front Cardiovasc Med. 2022 Jan 17;8:786943. doi: 10.3389/fcvm.2021.786943 (PMC8801497; doi:10.3389/fcvm.2021.786943)

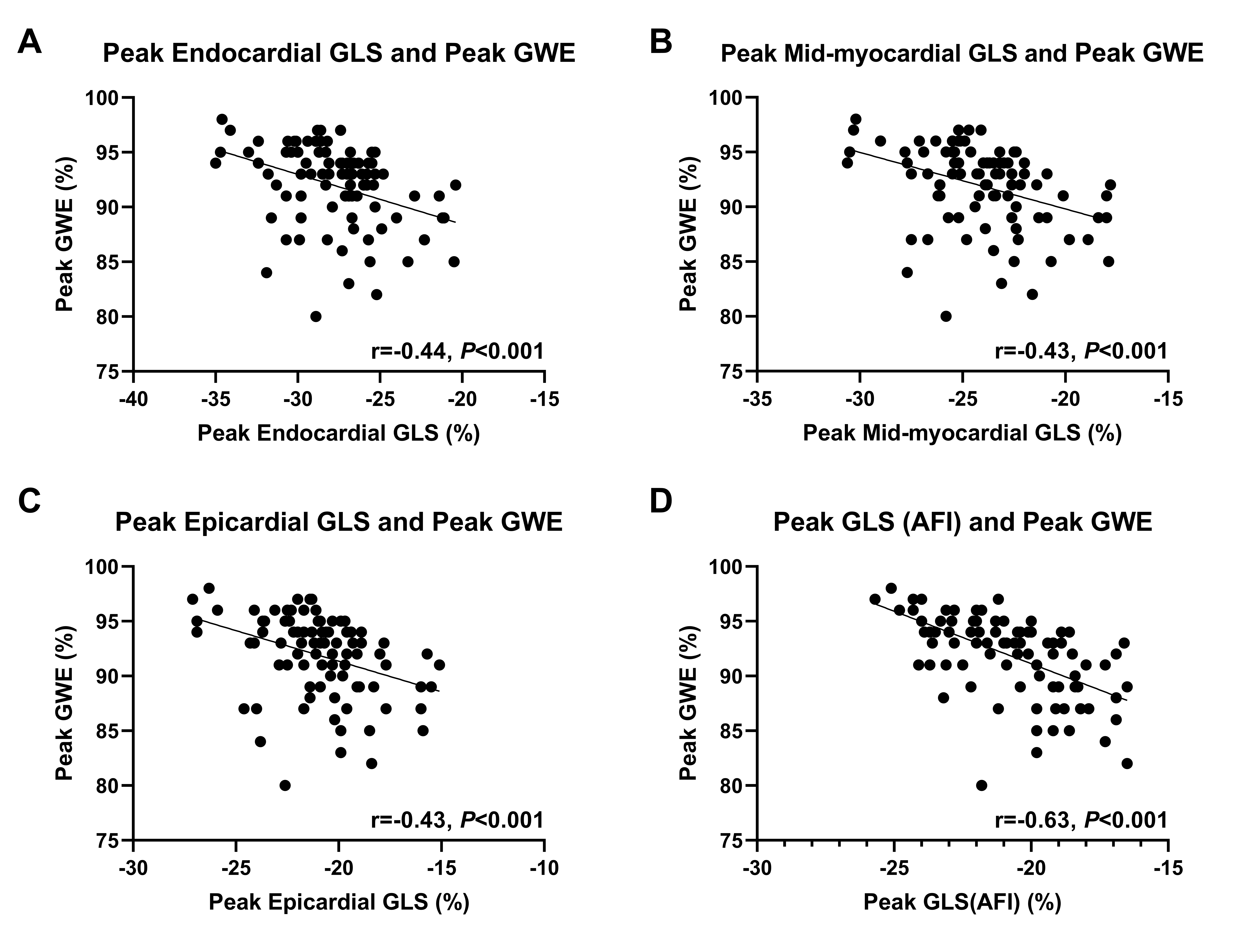

Supplement: Supplementary Figure 1 — Correlation between GWE and layer-specific strain, GLS (AFI) at peak exercise. (A) Correlation between peak GWE and peak endocardial GLS. (B) Correlation between peak GWE and peak mid-myocardial GLS. (C) Correlation between peak GWE and peak epicardial GLS. (D) Correlation between peak GWE and peak GLS (AFI). GLS, global longitudinal strain; GWE, global myocardial work efficiency; AFI, automated function imaging. [file Image_1.TIF]

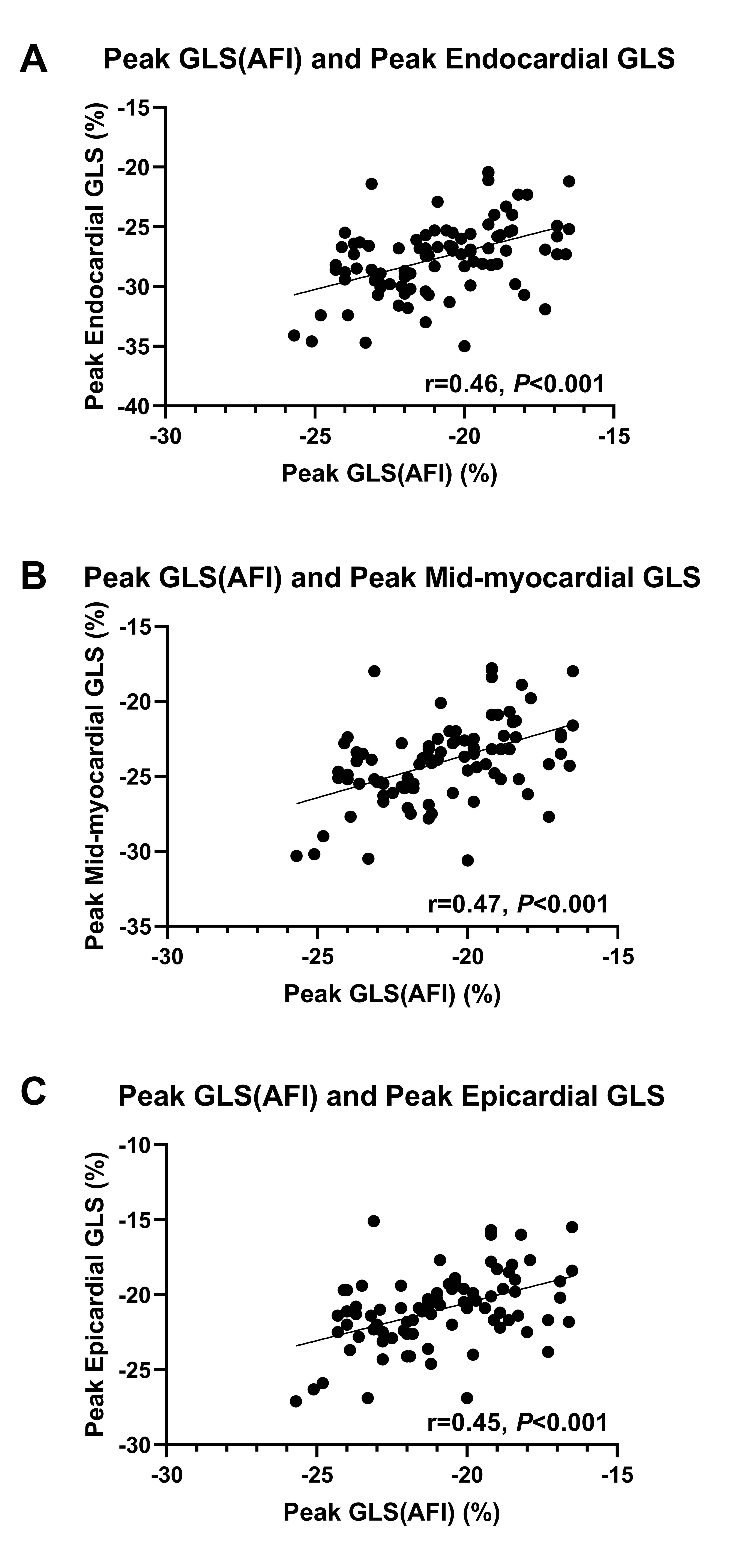

Supplement: Supplementary Figure 2 — Correlation between layer-specific strain and GLS (AFI) at peak exercise. (A) Correlation between peak endocardial GLS and peak GLS (AFI). (B) Correlation between peak mid-myocardial GLS and peak GLS (AFI). (C) Correlation between peak epicardial GLS and peak GLS (AFI). GLS, global longitudinal strain; AFI, automated function imaging. [file Image_2.TIF]
