# Supplementary material for: Comparison of Myocardial Layer-Specific Strain and Global Myocardial Work Efficiency During Treadmill Exercise Stress in Detecting Significant Coronary Artery Disease
Source: Front Cardiovasc Med. 2022 Jan 17;8:786943. doi: 10.3389/fcvm.2021.786943 (PMC8801497; doi:10.3389/fcvm.2021.786943)
Supplement: Supplementary Table 1 — Intra- and inter-observer variabilities for peak layer-specific strain parameters and peak GWE. ICC, Intraclass correlation coefficient; CI, confidence interval; CV, coefficient of variation; GLS, global longitudinal strain; GWE, global myocardial work efficiency. [file Table_1.docx]

**Supplementary TABLE 1** Intra- and Interobserver variabilities for peak layer-specific strain parameters and peak GWE

| Parameters | Intraobserver | |  | Interobserver | |
| --- | --- | --- | --- | --- | --- |
|  | ICC [95%CI] | CV (%) |  | ICC [95%CI] | CV (%) |
| Peak endocardial GLS, % | 0.885(0.664-0.961) | -3.25 |  | 0.883(0.692-0.959) | -3.63 |
| Peak mid-myocardial GLS, % | 0.916(0.775-0.971) | -2.64 |  | 0.854(0.618-0.949) | -3.91 |
| Peak epicardial GLS, % | 0.871(0.657-0.955) | -3.28 |  | 0.793(0.483-0.926) | -4.64 |
| Peak GWE, % | 0.907(0.751-0.967) | 0.88 |  | 0.875(0.625-0.958) | 1.09 |

ICC, Intraclass correlation coefficient; CI, confidence interval; CV, coefficient of variation; GLS, global longitudinal strain; GWE, global myocardial work efficiency.
